# Adult-Onset Still’s Disease as an Uncommon Cause of Fever of Unknown Origin

**DOI:** 10.7759/cureus.112922

**Published:** 2026-07-18

**Authors:** Yereni Aileen Andujar Sanchez, Lucero Moran, Diego Alexis Belmontes Gutiérrez, Pedro Antonio Moreno Velazco, Danya Michelle Isais Moreno

**Affiliations:** 1 Internal Medicine, Hospital General Universitario de Torreón Dr. Joaquin del Valle Sánchez, Torreón, MEX

**Keywords:** adult-onset still’s disease, biological therapy, fever of unknown origin, hyperferritinemia, interleukin-6

## Abstract

Adult-onset Still’s disease (AOSD) is a rare systemic autoinflammatory disorder characterized by fever, rash, arthritis, and hyperferritinemia, often presenting as a diagnostic challenge due to its nonspecific manifestations. We report the case of a 35-year-old previously healthy male who presented with a 20-day history of pruritic erythematous skin lesions, high-grade fever, severe odynophagia, and symmetric inflammatory polyarthritis. Laboratory evaluation revealed leukocytosis with neutrophilia, elevated acute-phase reactants, liver enzyme abnormalities, and hyperferritinemia (1,050 ng/mL). Infectious, autoimmune, and neoplastic causes were excluded. During hospitalization, the patient developed chest pain with electrocardiographic changes and elevated troponin levels, consistent with acute pericarditis. Skin biopsy demonstrated nonspecific inflammatory dermatitis. A diagnosis of AOSD was established based on clinical findings and Yamaguchi criteria. Initial treatment with systemic corticosteroids resulted in partial improvement; however, early relapse required escalation to tocilizumab, achieving complete clinical remission at two months. This case highlights the importance of considering AOSD in patients with fever of unknown origin and elevated inflammatory markers, as well as the role of early targeted therapy in improving outcomes.

## Introduction

Adult-onset Still’s disease (AOSD) is a rare systemic inflammatory condition considered part of the autoinflammatory disease spectrum. It is clinically characterized by high spiking fever, evanescent salmon-colored rash, arthritis or arthralgia, odynophagia, hyperferritinemia, and neutrophilic leukocytosis [1-3].

The etiology of AOSD remains unknown; however, a complex interaction between genetic predisposition (human leukocyte antigen (HLA) and non-HLA variants) and environmental factors, such as viral or bacterial infections acting as immunological triggers, has been proposed [4-7]. Epidemiologically, AOSD has an estimated incidence ranging from 0.16 to 0.4 cases per 100,000 persons per year, without significant sex differences, although some reports suggest slight female predominance. The typical age of onset ranges between 16 and 35 years [1-3,6]. The clinical presentation is heterogeneous, often leading to delayed diagnosis, reported in some cases up to four years [6]. 

From a pathophysiological perspective, AOSD involves immune dysregulation with predominant activation of the innate immune system, particularly macrophages and neutrophils, resulting in an exaggerated inflammatory cascade or “cytokine storm.” Key cytokines include IL-1β, IL-6, IL-18, TNF-α, and IFN-γ, along with molecules such as S100, CD163, and damage-associated molecular patterns (DAMPs) that activate the NLRP3 inflammasome [5,7-9]. This leads to systemic manifestations, multiorgan involvement, and risk of severe complications, such as macrophage activation syndrome (MAS), acute respiratory distress syndrome (ARDS), disseminated intravascular coagulation (DIC), and organ failure [2,7,9].

Three clinical patterns are recognized: monocyclic self-limited, polycyclic (relapsing-remitting), and chronic articular destructive disease. The latter may mimic seronegative rheumatoid arthritis and is associated with elevated IL-6, TNF-α, and IL-17 levels, whereas severe systemic forms correlate with elevated IL-18, IL-1β, and IFN-γ [6-9].

Diagnosis is primarily clinical and requires exclusion of infections, malignancies, and other autoimmune diseases. The most widely used criteria are those proposed by Yamaguchi (1992), which include high fever, evanescent rash, arthralgia, and leukocytosis as major criteria, and odynophagia, lymphadenopathy, liver dysfunction, and negative autoimmune serologies as minor criteria [6]. Serum ferritin, especially levels exceeding 3,000 ng/mL, has high diagnostic value and correlates with inflammatory activity [2-3,6].

Conventional treatment includes nonsteroidal anti-inflammatory drugs (NSAIDs) and systemic corticosteroids as first-line therapy. In refractory or steroid-dependent cases, disease-modifying antirheumatic drugs (DMARDs), such as methotrexate, are used. In recent years, biologic therapies targeting IL-1 (anakinra and canakinumab), IL-6 (tocilizumab), and, more recently, JAK inhibitors (tofacitinib and baricitinib) have shown significant efficacy, particularly in severe or systemic forms [5-9].

In summary, AOSD is a low-prevalence condition with high diagnostic and therapeutic complexity. Its polymorphic clinical presentation, lack of specific biomarkers, and potential progression to severe disease require a high index of suspicion, multidisciplinary evaluation, and individualized management.

## Case presentation

We report the case of a 35-year-old previously healthy male with a history of tobacco use who developed a subacute clinical picture 20 days prior to hospitalization. Initially, he presented with non-infiltrated erythematous skin lesions with irregular borders located on the trunk, abdomen, and thighs, associated with intense pruritus but no pain or temperature changes.

Subsequently, he developed severe odynophagia without improvement despite treatment with anti-inflammatory drugs and corticosteroids. By day seven, he developed fever up to 39 °C of unknown origin, accompanied by constitutional symptoms and symmetrical inflammatory polyarthritis involving both large and small joints, with severe pain (Visual Analog Scale (VAS) 10/10), joint swelling, functional limitation, and no morning stiffness.

Despite multiple outpatient treatments with antibiotics and glucocorticoids, the symptoms persisted, and the patient was hospitalized on day 18 for further evaluation. Physical examination revealed difficulty walking due to joint pain, symmetric erythematous skin lesions on the thighs, and swelling of multiple joints, including the shoulders, elbows, wrists, knees, and left ankle, without overt local inflammatory signs such as warmth or erythema.

Initial laboratory studies showed leukocytosis with neutrophilia, elevated C-reactive protein at 67 mg/L (reference range, <20 mg/L), and an elevated erythrocyte sedimentation rate of 39 mm/h (reference range, <20 mm/h). Liver enzymes were also elevated, including alanine aminotransferase (ALT) at 95 U/L (reference range, <35 U/L), gamma-glutamyl transferase (GGT) at 103 U/L (reference range, 12-43 U/L), and lactate dehydrogenase (LDH) at 323 U/L (reference range, 120-240 U/L). Viral serologies and rheumatologic markers were negative, including rheumatoid factor <20 IU/mL (negative, <20 IU/mL), creatine kinase <20 U/L (reference range, 30-170 U/L), and negative serologic tests for HIV, hepatitis B virus, and hepatitis C virus. Serum ferritin was elevated at 1,050 ng/mL (reference range, 10-300 ng/mL).

**Figure 1:**
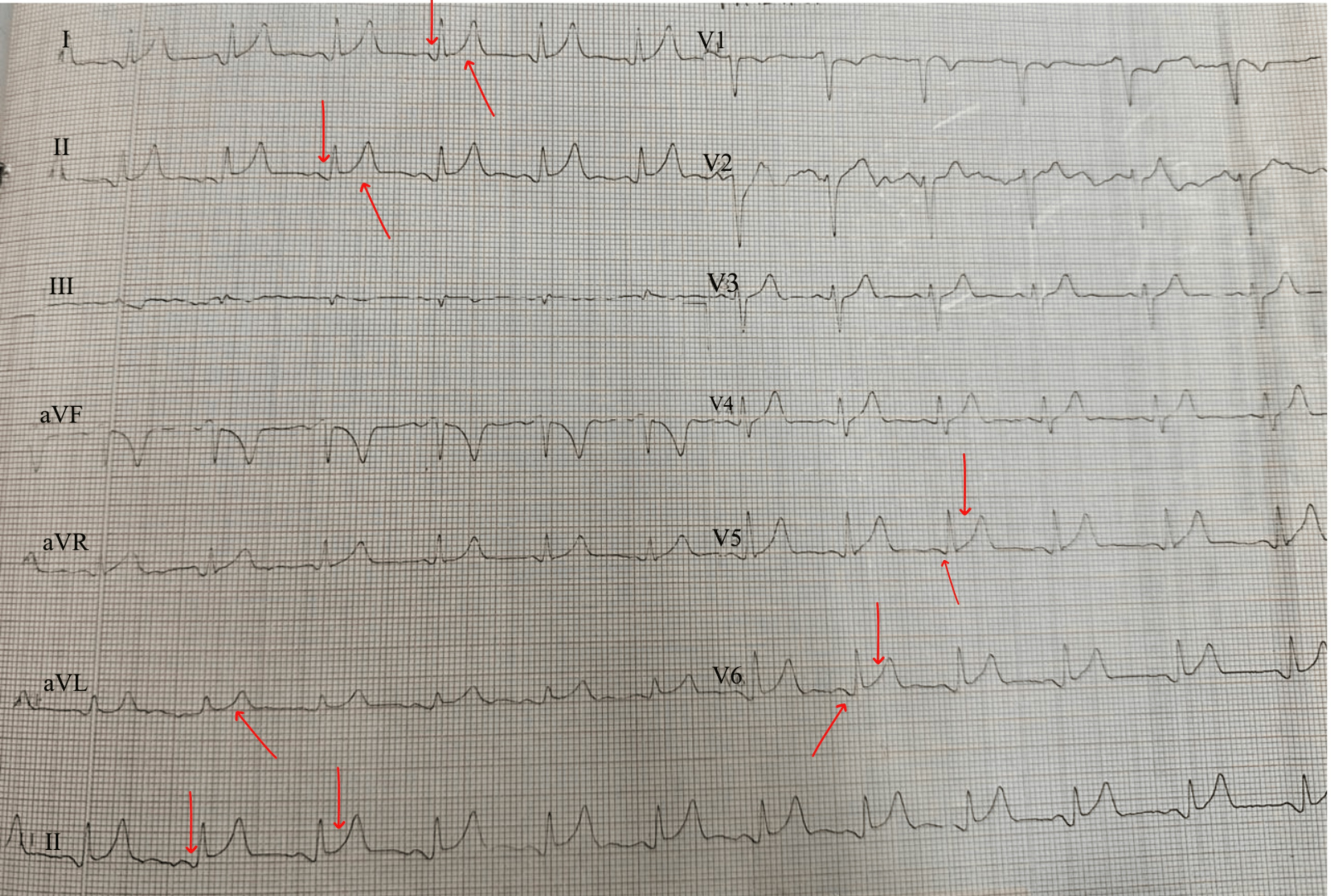
Acute pericarditis pattern The 12-lead electrocardiogram reveals sinus tachycardia with diffuse ST-segment elevation, characterized by a concave morphology in precordial leads V2 through V6 and lateral leads I and aVL. These changes are accompanied by generalized PR-segment depression, a configuration suggestive of Spodick's Stage I acute pericarditis.

During hospitalization, the patient reported diaphoresis, palpitations, and chest pain. ECG (Figure 1) showed diffuse concave ST elevation with peaked T waves, and elevated troponin I levels (0.7 and 1.29 ng/mL), suggestive of acute pericarditis in the context of systemic inflammation.

Antinuclear antibodies were negative. Skin biopsy revealed superficial perivascular dermatitis with lymphocytic and polymorphonuclear infiltrate without epidermal changes.

Based on clinical features, negative autoantibodies, and exclusion of other causes, a diagnosis of AOSD was established. Treatment was initiated with methylprednisolone 1 g pulse dose and indomethacin 50 mg, followed by prednisone 25 mg daily, colchicine 1 mg daily, and fexofenadine 180 mg daily.

At 24 hours, ferritin decreased to 864 ng/mL (reference range, 10-300 ng/mL). At the 15-day follow-up, the patient relapsed with generalized rash, requiring adjustment of treatment with methylprednisolone 80 mg single dose, prednisone 30 mg every 12 hours, and initiation of tocilizumab.

At the two-month follow-up, the patient showed complete clinical resolution, with improvement in rash and joint pain.

## Discussion

AOSD is a rare systemic autoinflammatory disease with complex diagnosis, classically presenting with fever, rash, and arthralgia. Differential diagnosis is particularly challenging in cases with nonspecific symptoms or atypical organ involvement, as in our patient.

In its 2022 recommendations, the European Alliance of Associations for Rheumatology (EULAR) emphasizes that AOSD should be considered in the differential diagnosis of fever of unknown origin when markedly elevated acute-phase reactants and hyperferritinemia are present and infectious, neoplastic, and autoimmune causes have been excluded [1].

This case represents a severe systemic onset, characterized by persistent fever >39 °C, disabling inflammatory polyarthritis, myalgias, atypical skin lesions, and pericardial involvement. This phenotype is associated with exaggerated innate immune activation mediated by cytokines, particularly IL-6, and increased risk of early relapse [10].

The diagnosis was established using the Yamaguchi criteria, recognized by the EULAR for their high sensitivity (96%) and specificity (92%) [11], along with hyperferritinemia and elevated inflammatory markers. Ferritin remains a relevant biomarker reflecting inflammatory activity, as it increases under cytokine regulation (IL-6, IL-1β, and TNF-α) independent of iron stores [12-13].

According to the EULAR 2022 guidelines, systemic corticosteroids are first-line therapy in moderate to severe disease, with pulse therapy indicated in organ involvement, as in this case. Early relapse after steroid tapering suggests steroid dependence, warranting early initiation of targeted biologic therapy.

Tocilizumab, an IL-6 receptor inhibitor, led to significant and sustained clinical improvement, allowing gradual steroid tapering, consistent with current evidence [14].

## Conclusions

Although AOSD is rare, its nonspecific presentation and variable course may lead to underrecognition. This case highlights the importance of early diagnosis and individualized treatment based on clinical phenotype and current guidelines.

AOSD should be considered in the differential diagnosis of patients with fever of unknown origin and persistent systemic symptoms, particularly in the presence of markedly elevated inflammatory markers. Early and appropriate management can significantly improve outcomes and prevent complications.

## References

[1] Castellano Cuesta JA, Corts Giner JR, Pastor Oliver FJ (2008). Adult-Onset Still's Disease [Article in Spanish]. Rheumatic diseases: SVR update.

[2] Ortega Carnicer J, Ceres F (2003). Adult-onset Still's disease and systemic inflammatory response syndrome. Role of blood ferritin [Article in Spanish]. Med Intensiva.

[3] Alarcón Cedeño N, Sánchez Cantos Y, Barcia Cansino A (2024). Adult-onset Still's disease (AOSD): a diagnostic challenge. Case report. Reumatología al Día.

[4] Sfriso P, Bindoli S, Galozzi P (2018). Adult-onset Still's disease: molecular pathophysiology and therapeutic advances. Drugs.

[5] Ma Y, Meng J, Jia J (2021). Current and emerging biological therapy in adult-onset Still's disease. Rheumatology (Oxford).

[6] Macovei LA, Burlui A, Bratoiu I (2022). Adult-onset Still's disease-a complex disease, a challenging treatment. Int J Mol Sci.

[7] Ruscitti P, Cantarini L, Nigrovic PA, McGonagle D, Giacomelli R (2024). Recent advances and evolving concepts in Still's disease. Nat Rev Rheumatol.

[8] Mitrovic S, Feist E, Fautrel B (2020). Adult-onset Still’s disease. Periodic and Non-Periodic Fevers.

[9] Masias-León Y, García DF, Ruíz-González CE (2023). Adult-onset Still's disease diagnosed late [Article in Spanish]. Med Int Méx.

[10] Ruscitti P, Cipriani P, Masedu F (2016). Adult-onset Still's disease: evaluation of prognostic tools and validation of the systemic score by analysis of 100 cases from three centers. BMC Med.

[11] Efthimiou P, Kontzias A, Hur P, Rodha K, Ramakrishna GS, Nakasato P (2021). Adult-onset Still's disease in focus: clinical manifestations, diagnosis, treatment, and unmet needs in the era of targeted therapies. Semin Arthritis Rheum.

[12] Mahroum N, Alghory A, Kiyak Z, Alwani A, Seida R, Alrais M, Shoenfeld Y (2022). Ferritin - from iron, through inflammation and autoimmunity, to COVID-19. J Autoimmun.

[13] Mehta B, Efthimiou P (2012). Ferritin in adult-onset still's disease: just a useful innocent bystander?. Int J Inflam.

[14] Fautrel B, Mitrovic S, De Matteis A (2024). EULAR/PReS recommendations for the diagnosis and management of Still's disease, comprising systemic juvenile idiopathic arthritis and adult-onset Still's disease. Ann Rheum Dis.

